# Supplementary material for: MetaRibo-Seq measures translation in microbiomes
Source: Nat Commun. 2020 Jun 29;11:3268. doi: 10.1038/s41467-020-17081-z (PMC7324362; doi:10.1038/s41467-020-17081-z)
Supplement: Supplementary file 10 — Supplementary Data 7 [file 41467_2020_17081_MOESM10_ESM.zip › File2/Confidence_High_Taxonomy/84181_out.krona.html]

Javascript must be enabled to view this page.

members
magnitude
magnitudeUnassigned
count
unassigned
taxon
rank

84181\_out

42

42
2
superkingdom

phylum
1239
42

class
186801
42

42
186802
1
order

SRS013098\_contig\_number\_contig-100\_8734.253505

41
family
216572

459786
41
genus

SRS022071\_contig\_number\_26345
1


SRS015217\_contig\_number\_26217SRS017916\_contig\_number\_contig-100\_619.113663SRS022713\_contig\_number\_5174SRS043411\_contig\_number\_763SRS043768\_contig\_number\_28946SRS048981\_contig\_number\_7010SRS049773\_contig\_number\_34534SRS051610\_contig\_number\_3080SRS054590\_contig\_number\_contig-100\_7806.7806SRS054956\_contig\_number\_11180SRS057478\_contig\_number\_contig-100\_7171.29917SRS076804\_contig\_number\_13723SRS077194\_contig\_number\_25327SRS077552\_contig\_number\_9741SRS077641\_contig\_number\_13533SRS098655\_contig\_number\_10998SRS103987\_contig\_number\_36446SRS1041036\_contig\_number\_contig-100\_1366.1367SRS1041136\_contig\_number\_23013SRS104311\_contig\_number\_42529SRS1054716\_contig\_number\_2869SRS1055049\_contig\_number\_562SRS142712\_contig\_number\_715SRS143342\_contig\_number\_21500SRS143991\_contig\_number\_27042SRS144362\_contig\_number\_31677SRS147139\_contig\_number\_43643SRS148159\_contig\_number\_contig-100\_17900.204160
species
2109687
28

876091

SRS013098\_contig\_number\_48343SRS015486\_contig\_number\_contig-100\_3780.3781SRS049900\_contig\_number\_contig-100\_14751.98927SRS097920\_contig\_number\_30857SRS097958\_contig\_number\_23313SRS100021\_contig\_number\_9810SRS1055099\_contig\_number\_6633SRS144297\_contig\_number\_contig-100\_31246.31247SRS146764\_contig\_number\_26701SRS147346\_contig\_number\_contig-100\_3754.352548SRS148721\_contig\_number\_43780SRS893342\_contig\_number\_contig-100\_11239.11240
species
12
